# Impact of Gender on Chronic Complications in Participants With Type 2 Diabetes: Evidence From a Cross‐Sectional Study

**DOI:** 10.1002/edm2.488

**Published:** 2024-05-08

**Authors:** Kiavash Mokhtarpour, Amirhossein Yadegar, Fatemeh Mohammadi, Seyedeh Nazanin Aghayan, Seyed Arsalan Seyedi, Soghra Rabizadeh, Alireza Esteghamati, Manouchehr Nakhjavani

**Affiliations:** ^1^ Endocrinology and Metabolism Research Center (EMRC), Vali‐Asr Hospital Tehran University of Medical Sciences Tehran Iran

**Keywords:** coronary artery disease, diabetes complications, diabetic retinopathy, gender difference

## Abstract

**Introduction:**

This study aimed to assess and compare the prevalence of diabetes complications between men and women with Type 2 diabetes (T2D), as well as how gender relates to these complications.

**Methods:**

In this cross‐sectional study, complications of diabetes, including coronary artery disease (CAD), retinopathy, neuropathy and diabetic kidney disease (DKD), were evaluated in 1867 participants with T2D. Additionally, baseline characteristics of the individuals, including anthropometric measurements, metabolic parameters and the use of dyslipidaemia drugs and antihyperglycaemic agents, were assessed. Gender differences in complications were examined using the chi‐squared test. Multivariate logistic regression was employed to investigate the relationship between gender and T2D complications, with and without adjusting for the characteristics of the studied population.

**Results:**

In the studied population, 62.1% had at least one complication, and complications were 33.5% for DKD, 29.6% for CAD, 22.9% for neuropathy and 19.1% for retinopathy. The prevalence of CAD and neuropathy was higher in men. However, DKD and retinopathy were more prevalent among women. Odds ratios of experiencing any complication, CAD and retinopathy in men compared with women were 1.57 (95% CI: 1.27–2.03), 2.27 (95% CI: 1.72–2.99) and 0.72 (95% CI: 0.52–0.98), respectively, after adjusting for demographic factors, anthropometric measures, metabolic parameters and the consumption of dyslipidaemia drugs and antihyperglycaemic agents.

**Conclusion:**

The prevalence of diabetes complications was significantly higher in men with diabetes, highlighting the need for better treatment adherence. CAD was associated with the male gender, whereas retinopathy was associated with the female gender. Men and women with diabetes should be monitored closely for CAD and retinopathy, respectively, regardless of their age, diabetes duration, anthropometric measures, laboratory findings and medications.

## Graphical abstract

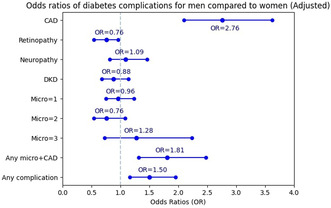

## 1 Introduction

According to the International Diabetes Federation (IDF), the prevalence of diabetes in 20‐ to 79‐year‐olds was 10.5% or 536.6 million people worldwide in 2021, causing the healthcare system to spend 966 billion USD on controlling diabetes [1]. By 2045, this number will increase to 12.2% or 783.2 million, and expenses will likely rise to 1054 billion USD [1]. Moreover, nearly 193 million individuals may have undiagnosed diabetes [1]. A significant share of over 90% of participants with diabetes have Type 2 diabetes (T2D), which can lead to microvascular and macrovascular complications [2]. It is widely known that complications of T2D tend to appear over time and menace health and quality of life, especially when diabetes is unmanaged. The most prevalent complications include cardiovascular disease (CVD), retinopathy, neuropathy and diabetic kidney disease (DKD) [3].

The global prevalence of diabetes in men is 10.8%, which is 0.6% more than in women [1]. All‐cause mortality rates decreased from 42.6 to 24.4 annual deaths per 1000 persons in men with diabetes between 1971 and 2000 in the US population [4]. However, these rates in the same period increased from 18.4 to 25.9 annual deaths per 1000 persons in women with diabetes, which indicates better progress in mortality reduction rates in men than in women [4]. Previous research has shown discrepancies between genders in the risk of experiencing micro‐ and macrovascular diabetes complications. Several studies reported a higher risk of CVD, non‐alcoholic fatty liver disease (NAFLD), retinopathy, neuropathy and diabetic nephropathy in men with diabetes [5, 6, 7, 8]. On the contrary, other studies showed that the risk of kidney disease, cancer, coronary heart disease (CHD) and retinopathy are higher among women with diabetes [9, 10, 11, 12]. Furthermore, research on gender differences in diabetes and its complications is limited, and recommendations are not specified for genders.

This study aimed to assess and compare the prevalence of various diabetes complications in men and women with T2D and examine the associations between gender and the risk of experiencing these complications.

## 2 Methods and Research Design

### 2.1 Study Design

In this cross‐sectional study, individuals with T2D referred to the diabetes clinic of a tertiary hospital affiliated with Tehran University of Medical Sciences were evaluated between January 2017 and July 2022. Diabetes among referred individuals was diagnosed according to the American Diabetes Association (ADA) criteria [13]. Because of the small sample size, individuals with current or past smoking history were excluded from the analysis. Therefore, 1867 participants with T2D were included in the study. The complications of diabetes were investigated in total and among women and men. The population was relatively homogenous, consisting of middle‐class individuals with education levels ranging from middle to high school. They had access to healthcare facilities and were covered by insurance. The Research Ethics Committee of Tehran University of Medical Sciences approved this study. All participants provided their written informed consent before entering the study. Additionally, the ethical principles of the Declaration of Helsinki were observed during the process.

### 2.2 Data Collection

Well‐trained examiners measured demographic features and anthropometric parameters, including age, duration of diabetes, medications in use, height, weight, waist circumference (WC) and hip circumference (HC) for each participant. A complete medical history was collected, including any record of acute or chronic diseases or diabetes complications, medications and smoking history. Weight was measured using a digital scale (Tefal PP1100), with the participant wearing light clothing and no shoes (nearest to 0.1 kg). Height was also measured using measuring tape while the participant was asked to stand straight against the wall with their feet together (most comparable to 0.1 cm). Body mass index (BMI) was calculated by weight in kilograms divided by height in metres squared. WC was measured by non‐elastic tape at the middle point between the lower borders of the rib cage and the iliac crest (rounded to the nearest 0.1 cm) [14]. A non‐elastic tape measure was placed at the widest part of the hips while the participant was standing with their feet together to measure HC (nearest to 0.1 cm). The waist‐to‐hip ratio (WHR) was calculated by dividing WC by HC.

Trained nurses measured blood pressure twice (5 min apart) after at least 10 min of resting using a calibrated Omron M7 digital sphygmomanometer (Hoofddorp, the Netherlands) and a properly sized cuff. Blood pressure ≥140/90 mmHg or being on therapy for hypertension was considered as hypertension [15]. Insulin, creatinine, haemoglobin A1c (HbA1c), fasting blood glucose (FBG), total cholesterol (TC), triglycerides (TG), low‐density lipoprotein cholesterol (LDL‐C) and high‐density lipoprotein cholesterol (HDL‐C) were measured in venous blood samples of each participant after about 12 h of fasting. Two‐hour postprandial blood glucose (2hpp) was analysed in collected specimens from 2 h after the beginning of an adequate breakfast. The glucose oxidase method (utilising Parsazmun Auto Analyzer, BT‐3000(plus); Biotechnica) was employed to measure FBG and 2hpp. HbA1c was assessed through high‐performance liquid chromatography using the DS5 instrument from DREW, England. TC, TG, LDL‐C, HDL‐C and creatinine were quantified using enzymatic techniques with the Parsazmun Auto Analyzer, BT‐3000(plus), Biotechnica. Insulin levels were determined via the chemiluminescence method employing the IMMULITE 2500 instrument from SIEMENS, provided by Immunotech. Spot urine samples in the early morning in a fasting state were collected from each participant to calculate the urine albumin–creatinine ratio (uACR) by dividing the urine albumin (mg/dL) by the urine creatinine (g/dL). In the usual protocol, patients were directed to gather their urine in containers supplied with boric acid from 7 am to 7 am the following day. Albumin and creatinine levels were measured in each urine sample (24‐h collections and spot urine samples). Urinary albumin excretion (UAE) was measured using the latex turbidimetric immunoassay method with the DAKO package from Glostrop, Denmark. Urinary creatinine levels were determined using the Jaffe colorimetric assay and an automated system (Liasys, Roma, Italy) with commercial kits (Pars Azmun, Tehran, Iran). The estimated glomerular filtration rate (eGFR) was determined using the Chronic Kidney Disease Epidemiology Collaboration equation [16]. Homeostatic model assessment of insulin resistance (HOMA‐IR) was calculated using the formula: HOMA‐IR = [glucose (nmol/L) × insulin (mU/mL)/22.5] [17]. BMI is categorised as follows: underweight (BMI < 18.5 kg/m^2^), normal (18.5 kg/m^2^ ≤ BMI < 25 kg/m^2^) and overweight and obese (BMI ≥25 kg/m^2^) [18]. The cut‐off values of other anthropometric measurements in Iranian adults are 98 cm in males and 84 cm in females for WC [19], and 0.87 in males and 0.78 in females for WHR, determining overweight and obesity [19]. As ADA recommends, clinical targets for blood lipid profile parameters are a TG level of <150 mg/dL, an LDL‐C level of <100 mg/dL and an HDL‐C level of ≥40 mg/dL in men and ≥50 mg/dL in women [20]. The abnormal FBG, HbA1c and eGFR range are ≥126 mg/dL, ≥ 6.5% and ≤ 84.8 mL/min/1.73 m^2^, respectively [20, 21]. Serum creatinine level with normal kidney function is expected to be between 0.6–1.2 and 0.5–1.1 mg/dL in males and females, respectively [22]. In the case of HOMA‐IR, the optimal cut‐off value for IDF‐defined metabolic syndrome in Iranian diabetic individuals is 4.325 [23].

### 2.3 Diabetes Complications

Coronary artery disease (CAD) was determined by medical history, including percutaneous coronary intervention, coronary artery angiography, coronary artery bypass graft, cardiac care unit admission and myocardial infarction. Additionally, the atherosclerotic cardiovascular disease (ASCVD) risk was calculated for those without a medical history. Those with a risk score of 5% or higher based on the ACC/AHA guideline for estimating a 10‐year ASCVD risk score [24] and not categorised as low risk were chosen for additional evaluations and tests. The selected participants were referred to a cardiologist and underwent non‐invasive tests like transthoracic echocardiography, exercise tolerance test or myocardial perfusion scan. The positive results in any of the three mentioned tests, which the same cardiologist interpreted, were evidence of a CAD diagnosis [25, 26, 27]. In the case of retinopathy, all of the participants were referred to an ophthalmologist. Under the ophthalmologist's advisory, the participants underwent a dilated fundus examination and optical coherence tomography. In the retinal examination, findings such as microvascular abnormalities (haemorrhages or microaneurysms, cotton–wool spots and increased vascular permeability), venous calibre abnormalities, vascular closure, diabetic macular oedema (from some apparent retinal thickening or hard exudates in the posterior pole [mild] to retinal thickening or hard exudates involving the centre of the macula [severe]), retinal neovascularisation, vitreous haemorrhage or preretinal haemorrhage were considered as diabetic retinopathy [28]. Data on neuropathy were collected through the completion of Michigan Neuropathy Screening Instrument (MNSI) [29] by a general practitioner or medical history of any diabetes‐related neurological condition, such as severe pain or numbness in the legs, feet or hands that led to limb ulcerations or amputations. Participants without a medical history at the clinic who scored greater than 2 (out of a maximum of 10 points) on the clinical section of MNSI and also had abnormal neurological examinations were considered neuropathic. A neurologist performed a comprehensive neurological examination on all participants. This examination encompassed evaluations of sensory and motor function and nerve conduction. DKD was diagnosed through interpretation of creatinine and albumin levels in urine (random spot and 24 h) and blood samples by a nephrologist or a general practitioner (uACR ≥30 mg/g, reduced eGFR below 60 mL/min/1.73 m^2^ and persisting elevated albuminuria of >300 mg/day were considered positive [30]). Proteinuria and renal dysfunctions caused by other causes than diabetes, such as drug‐induced nephrotoxicity, urinary tract infections and obstructions, were excluded by taking history and performing examinations if necessary. In these cases, samples were repeated 3 months after eliminating the potential cause. The DKD diagnosis was confirmed by positive results in at least one of the three performed tests on urine samples. Moreover, the International Classification of Diseases, Tenth Revision (ICD‐10), was used to identify chronic microvascular complications. The codes E11.2, E11.3 and E11.4 were used for DKD, retinopathy and neuropathy, respectively.

### 2.4 Statistical Analysis

The normality of the data was assessed with the Shapiro–Wilk test. The mean ± standard deviation (SD), median and the first and third quartiles (Q1–Q3) were calculated. For parametric variables, one‐way analysis of variance (ANOVA) and *t*‐tests were conducted to compare means across different groups. Non‐parametric variables were analysed with the Mann–Whitney *U* and Kruskal–Wallis tests for median comparisons. Categorical variables were expressed as numbers and percentages. The chi‐squared test was utilised to analyse categorical variables. To explore the relationship between diabetes complications and gender, logistic regression was applied initially, and the model was then refined through four step‐by‐step adjustments. Model 1 considered age and the duration of diabetes. The second model incorporated BMI, hypertension, age and diabetes duration. The third model included variables, including HbA1c, HOMA‐IR, TG and TC, in addition to those previously discussed. The final model was adjusted for dyslipidaemia drugs, antihyperglycaemic medications and the variables considered in the previous models. Statistical significance was determined with a threshold of *p*‐value <0.05. Data were analysed using the SPSS software, version 24 (SPSS, Inc.). Figures were illustrated using Python version 3.12 with NumPy version 1.26 and Matplotlib version 3.8.1 libraries [31, 32].

## 3 Results

### 3.1 Baseline Characteristics

In this research, 1867 individuals were involved, and slightly over half (53.1%) were women. The average age in the total population was 59.8 ± 10.0 years, and the duration of diabetes was 12 (6–20) years in both genders. According to regional cut‐offs, the percentage of women with elevated WHR was significantly higher than men (99.6% over 97.8%; *p*‐value < 0.001). Notably, the median WHR in women was 0.14 higher than the optimal cut‐off value for overweight and obesity; however, this difference was 0.09 in men. Women had higher BMI levels than men by 29.9 kg/m^2^ over 27.6 kg/m^2^ (*p*‐value < 0.001). Additionally, hypertension affected more women than men (47.2% vs. 40.0%; *p*‐value = 0.002). On the contrary, average 2hpp and creatinine levels were significantly higher in men (*p*‐value < 0.001). However, in women, blood lipid profile parameters, including TG, TC, LDL‐C, HDL‐C and non‐HDL‐C, were significantly higher (*p*‐value < 0.001). eGFR was significantly higher in men (80.1 mL/min/1.73 m^2^) than in women (73.4 mL/min/1.73 m^2^) (*p*‐value < 0.001). Moreover, HOMA‐IR was significantly higher in women than in men by 3.43 over 3.32 (*p*‐value = 0.043). However, HOMA‐IR was below 4.325 (the optimal cut‐off value for IDF‐defined metabolic syndrome in Iranian diabetic individuals) in both genders. There was no significant difference in terms of age, duration of diabetes, SBP, DBP, FBG and HbA1c between men and women. Multiple drug therapy and consumption of any sulfonylurea were significantly more prevalent among men. Oppositely, atorvastatin consumption and metformin monotherapy were significantly more prevalent in women. No significant differences were found between genders regarding the usage of other medications, including insulin, rosuvastatin and fibrates (Table 1).

**TABLE 1:** Baseline characteristics of patients with Type 2 diabetes based on gender.

	Total	Women	Men	*P‐value*
Age (years)	59.8 ± 10.0	60.0 ± 9.3	59.6 ± 10.8	0.387
Duration of T2D (years)	12.0 (6–20)	12.0 (6–20)	12.0 (6–20)	0.974
WC (cm)	98.9 ± 9.1	98.5 ± 9.0	99.4 ± 9.3	**0.044**
HC (cm)	105.2 ± 7.9	106.9 ± 8.6	103.3 ± 6.4	**<0.001**
WHR	0.94 ± 0.05	0.92 ± 0.05	0.96 ± 0.05	**<0.001**
Elevated WHR (Women > 0.78, Men > 0.87)	1844 (98.8%)	988 (99.6%)	856 (97.8%)	**<0.001**
BMI (kg/m^2^)	28.8 ± 4.6	29.9 ± 4.9	27.6 ± 3.9	**<0.001**
SBP (mmHg)	130.7 ± 15.9	130.5 ± 15.5	130.8 ± 16.3	0.664
DBP (mmHg)	78.9 ± 8.0	78.8 ± 7.8	78.9 ± 8.1	0.703
Hypertension, *N* (%)	818 (43.8%)	468 (47.2%)	350 (40.0%)	**0.002**
FBG (mg/dL)	161.5 ± 55.3	160.1 ± 54.0	163.1 ± 56.7	0.245
HbA1c (%)	7.81 ± 1.51	7.78 ± 1.48	7.84 ± 1.54	0.464
2hpp (mg/dL)	225.5 ± 84.7	218.9 ± 82.3	232.9 ± 86.8	**<0.001**
Creatinine (mg/dL)	1.00 ± 0.22	0.93 ± 0.20	1.09 ± 0.22	**<0.001**
eGFR (mL/min/1.73 m^2^)	76.6 ± 17.1	73.4 ± 17.4	80.1 ± 16.0	**<0.001**
HOMA‐IR	3.38 (2.50–4.61)	3.43 (2.55–4.69)	3.32 (2.45–4.55)	**0.043**
TG (mg/dL)	149.0 (107–202)	155.0 (113–206)	140.0 (100–200)	**0.001**
TC (mg/dL)	175.6 ± 43.2	183.2 ± 43.5	167.0 ± 41.2	**<0.001**
LDL‐C (mg/dL)	97.9 ± 33.3	101.6 ± 34.9	93.7 ± 30.9	**<0.001**
HDL‐C (mg/dL)	44.9 ± 10.9	47.9 ± 11.1	41.5 ± 9.6	**<0.001**
Non‐HDL‐C (mg/dL)	130.7 ± 41.7	135.3 ± 42.9	125.5 ± 39.7	**<0.001**
Medication				
Dyslipidaemia drug, *N* (%)				
Atorvastatin	978 (52.4%)	543 (54.7%)	435 (49.7%)	**0.033**
Rosuvastatin	333 (17.8%)	182 (18.3%)	151 (17.3%)	0.545
Fibrates	46 (2.5%)	21 (2.1%)	25 (2.9%)	0.370
Antihyperglycaemic agents, *N* (%)				
Multiple drug therapy	961 (51.5%)	489 (49.3%)	472 (53.9%)	**0.046**
Insulin	21 (1.1%)	7 (0.7%)	14 (1.6%)	0.080
Metformin monotherapy	762 (40.8%)	446 (45.0%)	316 (36.1%)	**<0.001**
Any sulfonylurea	123 (6.6%)	50 (5.0%)	73 (8.3%)	**0.005**

*Note:* Data are presented as mean ± SD, median (Q1–Q3) or number (%). P‐value<0.05 is considered statistically significant are shown in bold.

Abbreviations: 2hpp, two‐hour postprandial blood glucose; BMI, body mass index; DBP, diastolic blood pressure; eGFR, estimated glomerular filtration rate; FBG, fasting blood glucose; HbA1c, haemoglobin A1c; HC, hip circumference; HDL‐C, high‐density lipoprotein cholesterol; HOMA‐IR, homeostatic model assessment for insulin resistance; LDL‐C, low‐density lipoprotein cholesterol; SBP, systolic blood pressure; TC, total cholesterol; TG, triglycerides; WC, waist circumference; WHR, waist‐to‐hip ratio.

### 3.2 Complications of Diabetes

Among the total population of the study, 29.6% had CAD. Men experienced CAD more than women, with a prevalence of 37.6% over 22.6% (*p*‐value < 0.001). Additionally, CAD was the most common complication in men (Table 2). In total, 19.1% of individuals experienced retinopathy. The prevalence of retinopathy was not significantly different between genders (women: 20.1%, men: 18.1%; *p*‐value = 0.289). Neuropathy affected 22.9% of total participants. The prevalence of neuropathy among men and women was 23.4% and 22.4%, respectively, which had no significant difference (*p*‐value = 0.619). DKD had the highest prevalence among the studied population (33.5%). The most common complication in women was DKD, and its prevalence was slightly higher than in men (32.9%). However, the difference was insignificant (*p*‐value = 0.623) (Table 2).

**TABLE 2:** Prevalence of chronic diabetes complications according to gender.

	Total	Women	Men	*P‐value*
CAD	553 (29.6%)	224 (22.6%)	329 (37.6%)	**<0.001**
Retinopathy	357 (19.1%)	199 (20.1%)	158 (18.1%)	0.289
Neuropathy	427 (22.9%)	222 (22.4%)	205 (23.4%)	0.619
DKD	626 (33.5%)	338 (34.1%)	288 (32.9%)	0.623
Number of microvascular complications				
1	576 (30.9%)	301 (30.3%)	275 (31.4%)	0.616
2	273 (14.6%)	160 (16.1%)	113 (12.9%)	0.057
3	96 (5.1%)	46 (4.6%)	50 (5.7%)	0.296
Any microvascular complication + CAD	338 (18.1%)	149 (15.0%)	189 (21.6%)	**<0.001**
Any complication	1160 (62.1%)	582 (58.7%)	578 (66.1%)	**0.001**

*Note:* Data are presented as number (%). P‐value<0.05 is considered statistically significant are shown in bold.

Abbreviations: CAD, coronary artery disease; DKD, diabetic kidney disease.

Microvascular complications, including retinopathy, neuropathy and DKD, affected 50.6% of the total population, 51.1% of women and 50.1% of men (*p*‐value = 0.676). The prevalence of one, two or three simultaneous microvascular complications in the total population was 30.9%, 14.6% and 5.1%, respectively. The prevalence of experiencing one or three microvascular complications at a time was higher in men, and the prevalence of two simultaneous microvascular complications was higher in women. Nonetheless, the difference in prevalence between genders was not significant (Table 2).

In the total population of the study, 62.1% of participants experienced at least one complication of diabetes. The prevalence of any complication was significantly higher in men (66.1%) than in women (58.7%) (*p*‐value = 0.001). Moreover, the prevalence of any microvascular complications and concurrent CAD was significantly higher in men than in women (21.6% vs. 15.0%; *p*‐value < 0.001) (Table 2).

### 3.3 Association Between Gender and Complications of Diabetes

**FIGURE 1:**
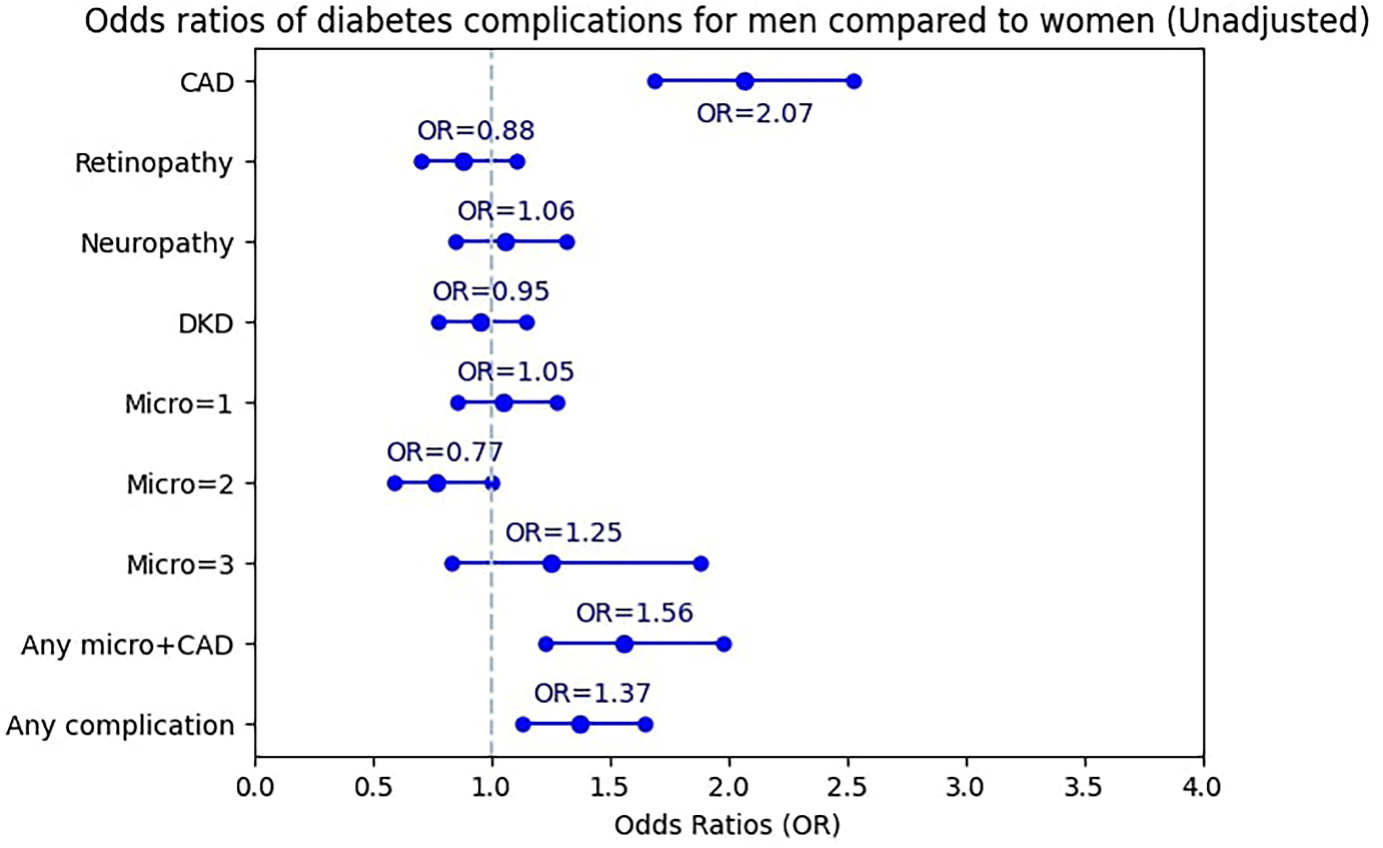
Unadjusted odds ratios (ORs) for various diabetes‐related complications. The blue markers represent OR, whereas error bars indicate the 95% confidence intervals.

**FIGURE 2:**
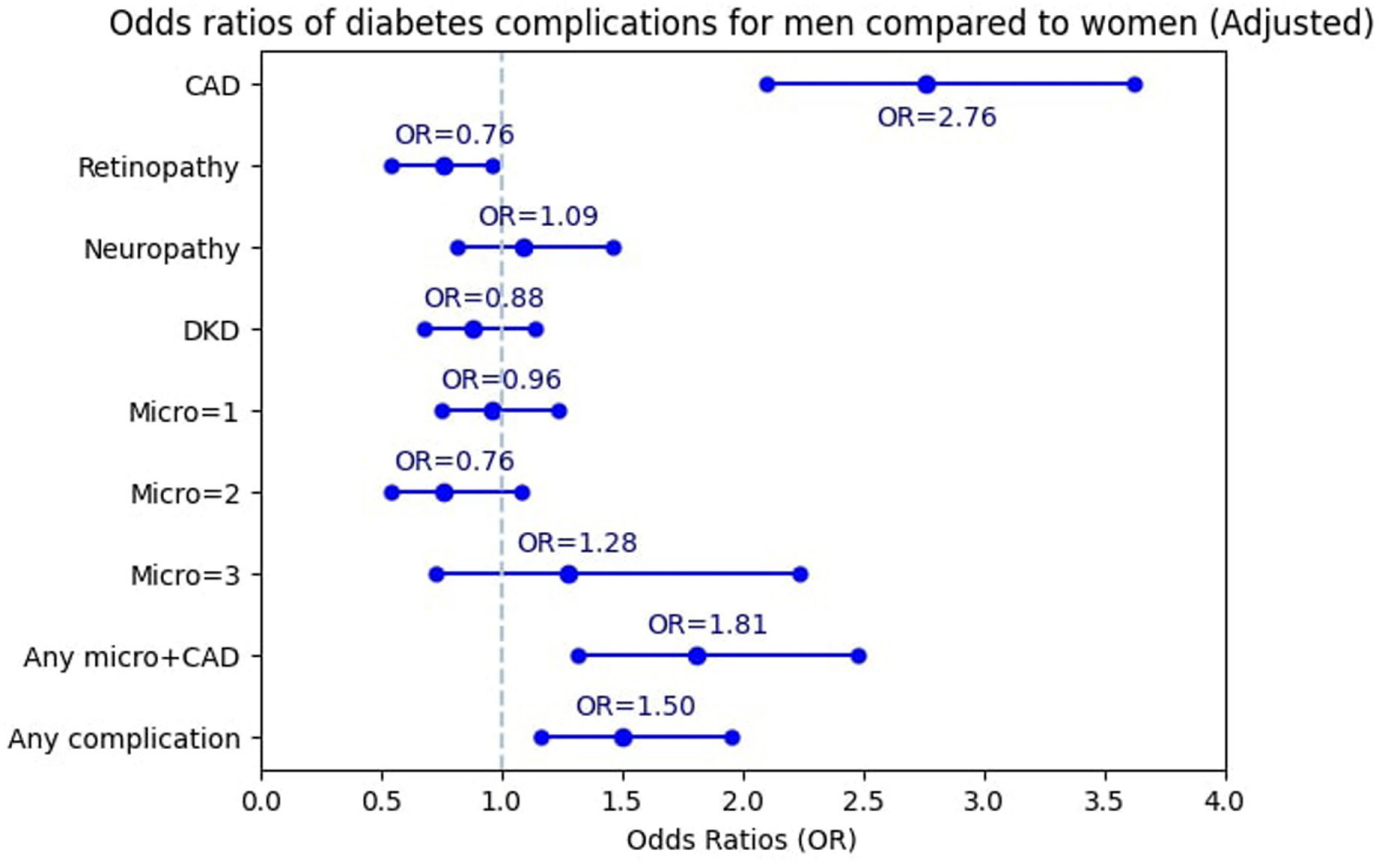
Adjusted odds ratios (ORs) for different diabetes complications. The blue markers indicate OR, whereas error bars indicate 95% confidence intervals.

Women were used as reference. The OR of experiencing CAD for men was 2.07 times more than for women before adjustment (95% CI: 1.69–2.53) (Figure 1). After full adjustment, it rose to 2.76 (95% CI: 2.10–3.62) (Figure 2). The OR of retinopathy in men was not significant in the unadjusted model. However, after adjusting for confounding factors, men had significantly lower odds of retinopathy than women (OR = 0.76; 95% CI: 0.54–0.97). The association between gender and neuropathy, DKD, and one, two or three simultaneous microvascular complications was not significant, either in the unadjusted or in the adjusted model. The odds of experiencing any microvascular complications and concurrent CAD for men was 1.56 times more than for women (95% CI: 1.23–1.98). Additionally, the significant association between gender and any microvascular complications and concurrent CAD in the adjusted model illustrated a 1.81 odds ratio (95% CI: 1.32–2.48) in men compared with women. The odds of experiencing any diabetes complication for men was 1.37 (95% CI: 1.13–1.65) times more than for women in the unadjusted model (Figure 1). After adjustment, the OR increased to 1.50 (95% CI: 1.16–1.95) (Figure 2).

## 4 Discussion

This study showed that 66.1% of men experienced diabetes complications, which was significantly higher than women (58.7%). Additionally, men were 1.50 times more likely to have at least one complication than women, regardless of any differences in demographic factors, anthropometric measures, laboratory findings and the use of dyslipidaemia and antihyperglycaemic medications. A similar study in India demonstrated a lower incidence of diabetes complications among women. They reported that the men had a higher prevalence of neuropathy (42.9% over 36.9%), retinopathy (36.0% over 26.3%) and nephropathy (25.0% over 23.3%). Furthermore, they demonstrated that men had a 1.8, 1.6 and 1.4 times higher chance of CVDs, retinopathy and neuropathy than women, suggesting that the male gender is a risk factor for these complications [33].

The higher prevalence of complications among men can be due to differences between men and women as aspects of genetic makeup, socio‐cultural habits, exposure to inappropriate lifestyle and excessive stress, nutrition, attitude to treatment and prevention of the disease, social roles and mental health [34, 35, 36, 37, 38]. Regarding genetic makeup differences, regulatory differences in blood glucose and insulin levels are heritable traits. When estimated separately, the heritability of T2D based on single nucleotide polymorphisms (SNPs) is notably higher in men than in women [39, 40]. This finding suggests that there are sex‐specific genetic influences on T2D risk because of specific genes or loci [41], which can also affect the development of diabetes complications. Additionally, sex‐specific differences in health behaviours, nutrition and physical activity have been observed. According to sex‐stratified health research data, women tend to be more inactive overall. However, they also tend to have healthier dietary habits, consuming more fruits and vegetables and less meat than men [37].

### 4.1 CAD

CAD mainly influenced the male gender in this study. The prevalence of CAD was significantly higher in men than in women (37.6% vs. 22.6%). A cohort study in the European population found that a higher percentage of individuals with CAD and T2D were men (68.3% were men) [42]. Oppositely, meta‐analyses from 64 cohort studies indicated that women with diabetes are 27% and 44% higher at risk of stroke and CHD than men with diabetes [11, 43, 44]. In several studies, women with diabetes, compared with men when treated similarly, were less likely to obtain goal values for cardiovascular risk factors and hyperglycaemia [45, 46]. Furthermore, it was found that women have higher cardiovascular risk factors prior to the onset of diabetes, which can be responsible for the greater CHD risk in women with diabetes than in men [47, 48].

In the current study, the percentage of elevated WHR in women was significantly higher than in men. It has been shown that WHR is associated with CAD morbidity, and it has a more substantial relation with CVD events than other anthropometric variables [49, 50]. Moreover, the prevalence of hypertension and mean TC, TG, LDL‐C and HDL‐C levels, which are directly associated with CAD risk [51, 52], were significantly higher in women. Oppositely, men had poorer glycaemic control and significantly higher 2hpp levels. However, after considering these discrepancies in baseline characteristics of both genders, men had a 2.76 times risk of experiencing CAD compared with women. This highlights the effect of the male gender as an independent risk factor for CAD in individuals with T2D. Similar to this analysis, previous studies showed more disrupted cardiometabolic profiles, including high BMI, hypertension and dyslipidaemia among women with diabetes [53, 54, 55, 56, 57]. However, there are disagreements regarding cardiovascular risk differences between genders. According to data from the UK Biobank on individuals with T2D, the excess risk of a cardiovascular event was approximately 50% higher in women than in men [58]. Conversely, an Indian study that specifically evaluated sex‐based differences of complications in individuals with diabetes showed a 1.8 times higher chance of CVDs in men with diabetes, which was in line with our findings [33].

CVD is the most prevalent adverse outcome of diabetes. Various biological and pathophysiological factors contribute to inherent sex differences in the incidence of CVD [43, 59]. These differences are responsible for variations in the prevalence of CVD events among men and women with diabetes in different countries. Sex steroid hormones, especially oestrogen in women, protect the cardiovascular system and protect them from CVD [60]. Low levels of total testosterone in men and extremes of bioavailable or unbound testosterone in women predicted coronary events [61]. It seems that after menopause and its following metabolic changes, women become more vulnerable to CVDs [62, 63, 64], which influences gender differences in the prevalence of heart diseases among individuals with T2D. Moreover, gender, as a social construct, can, directly and indirectly, influence individuals' cardiovascular risk by affecting biological markers such as blood pressure and lipid profile and pathways, including immune inflammation, oxidative stress and hormone production [65]. For instance, the early socialisation process encourages men to develop better physical activity [66] and women, as dominant caregivers, tolerate excessive stress, leading to anxiety and poor mental health, which are risk factors for CVD incidents [65]. On the contrary, several studies illustrated a lower health check uptake rate in men than in women [67, 68, 69].

### 4.2 Retinopathy

After adjustment for demographic factors, anthropometric measures, metabolic parameters and medications, this study found that the female gender is independently associated with retinopathy in T2D participants. The odds ratio of developing retinopathy in men compared with women was 0.76 after adjustment.

Sex seems to be a substantial factor in the incidence of diabetic retinopathy. However, the reasons behind gender‐based differences are unknown [70]. Previous studies indicated variations regarding the associations between sex and diabetic retinopathy. Several studies have shown that the female sex is an independent risk factor for retinopathy [12, 71, 72]; some depicted the male sex [6, 73, 74, 75, 76], and some detected no association [77, 78]. The variations might be due to differences in studied populations, including duration of diabetes, comorbidities, race, region and economic level, as well as differences in the study methods and designs [72].

In this study, a relationship between the female gender and retinopathy was found. According to a study in China on 12,766 participants, the female gender was independently associated with retinopathy in individuals whose T2D history was more than 10 years, whose ages were over 60 years, or who were in a relatively intermediate economic area [72]. Conversely, analysis of a cross‐sectional study in the United States on 1006 individuals with diabetes demonstrated the male gender as an independent risk factor for retinopathy with an odds ratio of 2.07 [73]. Biological variances, such as sex hormones, might affect retinal damage in diabetes [79]. One illustration of this is the role of fluctuating sex hormone levels in the advancement of DR during pregnancy [80]. Additionally, variations in inflammatory cytokine profiles based on gender could potentially impact the development and progression of DR [81]. For example, tumour necrosis factor‐alpha (TNF‐α) is an inflammatory cytokine, and recent findings showed that elevated levels of this cytokine can predict the presence of diabetic retinopathy [82]. Additionally, TNF‐α level positively correlates with HOMA‐IR and BMI in individuals with diabetes [83]. In the current study, HOMA‐IR and BMI were significantly higher among women, which can be one of the reasons for gender differences in retinopathy. Further investigations are required to evaluate the underlying mechanisms of the paradoxical results of gender differences regarding retinopathy in individuals with T2D.

### 4.3 DKD

DKD highly influenced both genders. DKD was the most prevalent complication in the total population, affecting 34.1% of women and 32.9% of men. In this study, no significant association was found between gender and DKD. Previous studies investigated the sex‐based differences in developing DKD, which discovered different results. A clinic‐based retrospective longitudinal study with a median follow‐up of 8.1 years in participants with T2D indicated a higher eGFR decline in women than in men (−3.5 ± 2.7% over −2.0 ± 2.2%) [84]. Oppositely, a cohort study with a median follow‐up duration of 5.7 years showed a higher glomerular filtration rate decline in men and introduced the male gender as an independent risk factor for renal function decline in T2D [85]. Eventually, the findings around the association between gender and DKD are controversial. The current and previous studies regarding gender and DKD differed in multiple factors, such as study design, sample size, baseline characteristics and regional discrepancies of the studied populations, such as lifestyle. In Kajiwara et al.'s study in Japan, women were significantly older and had higher systolic blood pressures than men. The prevalence of hypertension in women in Kajiwara et al.'s study was approximately 16% higher than in men, which is two times higher than the difference between genders in the current study. In De Hauteclocque et al.'s study in France, the prevalence of active smoking was significantly higher in men, and the age of the participants was approximately 5 years higher than the average age in the current study. Additionally, the association between gender and DKD in the current study is adjusted by age, duration of diabetes, hypertension and other factors that might affect this association.

According to Piani et al.'s review of 38 studies regarding sex‐related differences in DKD, men with either T1D or T2D seem to face an increased risk of DKD compared with premenopausal women [86]. Conversely, postmenopausal women exhibit a higher risk of developing DKD than both men and premenopausal women [86]. Several pathophysiologic mechanisms, including sex hormones, adiponectin levels, oxidative stress metabolism and water–electrolyte homeostasis and channels, impact these sex‐based differences, but exact mechanisms remain undiscovered [86]. For example, a decline in serum testosterone levels, observed in men with either T1D or T2D compared with healthy individuals, is linked to the development of macroalbuminuria [87, 88]. Additionally, oxygen consumption and production imbalance in the kidneys are critical pathogenetic features of many kidney diseases, including DKD, and there is evidence of higher degrees of oxidative stress in men than in women [89, 90]. Higher adiponectin concentrations are also found in women, suggesting a possible role of adiponectin in DKD sexual dimorphism, as it plays an essential role in inflammation and insulin sensitivity [91].

### 4.4 Neuropathy

In the current study, the prevalence of neuropathy was slightly higher in men than in women (23.4% over 22.4%), and the associations between genders and neuropathy were insignificant. A cross‐sectional study of 125 participants found no gender‐based differences in diabetic peripheral neuropathy [92]. On the contrary, two cross‐sectional studies on T1D and T2D individuals and a case–control study on 110 participants with diabetes indicated a higher incidence of neuropathy and distal symmetrical polyneuropathy in men [7, 93, 94]. Additionally, a study in a Cleveland, Ohio, hospital demonstrated a 4‐year earlier development of neuropathy in men than in women [95].

Overall, studies regarding gender differences in developing neuropathy in individuals with T2D are limited. The previous studies with contrary findings between gender and neuropathy compared with the current study had smaller samples, variations in types of diabetes and different study methods such as retrospective chart analysis and case–control study. Moreover, age and duration of diabetes are two critical factors in developing neuropathy [96]. The group of males and females in the current study had no significant difference in age and duration of diabetes, which can affect the association between gender and neuropathy.

### 4.5 Strength

The current study examined the complications of diabetes in women and men, both separately and combined. It also investigated the relationship between diabetes complications and sex regardless of influential factors such as age, duration of diabetes, BMI, hypertension, laboratory results and medications. This approach improves the gender‐specific point of view on diabetes complications.

### 4.6 Limitations

There were some limitations in the current study. First, the generalisability of the findings might be limited because the characteristics of participants who visit the diabetes clinic at a tertiary centre may not represent the overall population of people with diabetes in the community. In addition, unknown confounding variables, which we did not include in the analysis, may also affect the results. In addition, a larger sample size enrolment could augment the quality of the results.

## 5 Conclusion

In this cross‐sectional study, the prevalence of diabetes complications was significantly higher among men with diabetes, emphasising the necessity of better treatment adherence and lifestyle improvements in men. CAD was associated with the male gender, and retinopathy was associated with the female gender independent of their age, diabetes duration, anthropometric measures, laboratory findings and medications. This finding highlights the importance of better screening and follow‐up for CAD in men with diabetes and retinopathy in women with diabetes. Notably, further studies are required to enhance our comprehensive comprehension and to create tailored treatments that account for the inherent variations in pathophysiology between genders.

## Author Contributions

**Kiavash Mokhtarpour:** data curation (equal), investigation (equal), methodology (equal), writing–original draft (lead), writing–review and editing (equal). **Amirhossein Yadegar:** formal analysis (lead), methodology (equal), software (equal), visualization (equal), writing–review and editing (equal). **Fatemeh Mohammadi:** formal analysis (equal), methodology (supporting), software (equal), visualization (equal), writing–review and editing (supporting). **Seyedehnazanin Aghayan:** data curation (supporting), investigation (equal), writing–original draft (equal). **Seyed Arsalan Seyedi:** data curation (equal), formal analysis (equal). **Soghra Rabizadeh:** methodology (equal), supervision (equal), validation (equal). **Alireza Esteghamati:** conceptualization (equal), methodology (equal), supervision (equal). **Manouchehr Nakhjavani:** conceptualization (lead), methodology (lead), project administration (lead), supervision (equal), validation (equal), visualization (equal), writing–review and editing (lead).

## Ethics Statement

This study was conducted according to the Declaration of Helsinki principles and granted by the Tehran University of Medical Sciences ethics committee.

## Consent

Informed consent was obtained from all participants included in the study.

## Conflicts of Interest

The authors declare no conflicts of interest.

## Data Availability

The dataset used in this study is available upon request from the corresponding author.
